# Oxidative Stress and Endothelial Dysfunction: The Pathogenesis of Pediatric Hypertension

**DOI:** 10.3390/ijms26115355

**Published:** 2025-06-03

**Authors:** Kyle Backston, Jordan Morgan, Samipa Patel, Riddhima Koka, Jieji Hu, Rupesh Raina

**Affiliations:** 1 College of Medicine, Northeast Ohio Medical University, Rootstown, OH 44272, USA; 2 Solon High School, Solon, OH 44139, USA; 3 Department of Nephrology, Akron Nephrology Associates/Cleveland Clinic Akron General Medical Center, Akron, OH 44302, USA; 4 Department of Nephrology, Akron Children’s Hospital, Akron, OH 44308, USA

**Keywords:** pediatric hypertension, reactive oxygen species (ROS), endothelial dysfunction, oxidative stress, inflammation, chronic kidney disease

## Abstract

Pediatric hypertension is increasingly recognized as a complex condition shaped by both systemic and cellular factors, with oxidative stress emerging as a key driver of vascular dysfunction. In both their primary and secondary forms, reactive oxygen species (ROS) disrupt redox homeostasis, impair endothelial signaling, and promote inflammation and tissue remodeling. Metabolic dysregulation, renal pathology, and early-life stressors contribute to the accumulation of ROS through pathways involving NADPH oxidases, mitochondrial dysfunction, xanthine oxidase activity, and altered arginine metabolism. These mechanisms converge on the vasculature, diminishing nitric oxide bioavailability and promoting hypertensive phenotypes. Beyond disease initiation, redox imbalance influences the response to treatment, surgical outcomes, and long-term cardiovascular risk. By further elucidating these mechanisms, the complex relationship between oxidative stress, vascular biology, and blood pressure regulation in children may be more clearly defined and more effectively targeted in clinical management.

## 1. Introduction

The prevalence of hypertension in children and adolescents, estimated to be between 2 and 5%, has rapidly risen over several decades and is now recognized as a global issue [1,2,3,4,5,6]. Hypertension is characterized by an increase in peripheral vascular resistance and arterial stiffening and may be correlated with obesity, insulin resistance, and ventricular remodeling, which is indicative of early cardiovascular disease in pediatric populations [1,3,5,7]. Additionally, pediatric hypertension has major health implications that extend into adulthood. Hypertensive pediatric patients have a higher likelihood of being hypertensive in adulthood, increasing their long-term risk of complications such as stroke, cardiac events, and chronic kidney disease [1,5,7,8,9].

Guidelines for pediatric hypertension include the Fourth Report on the Diagnosis, Evaluation, and Treatment of High Blood Pressure in Children and Adolescents [10]. The most current revisions of hypertension guidelines were published by the European Society of Hypertension (ESH) in 2016 and the American Academy of Pediatrics (AAP) in 2017 [2,3,11]. Additionally, various publications exist globally to offer insights tailored to pediatric populations in various regions that reflect norms in growth patterns, average systolic pressures, and broader sociodemographic characteristics [12,13,14,15]. Across all guidelines, pediatric hypertension is defined as an elevation in systolic BP (SBP) or diastolic BP (DBP) that is greater than or equal to the 95th percentile by age, sex, and height, confirmed on three separate occasions [1,11,12,13,15]. Additionally, greater emphasis is now placed on distinguishing primary hypertension from secondary hypertension, the latter driven by an underlying disease process [1]. While these guidelines provide a structured approach to diagnosing and managing pediatric hypertension, understanding the underlying pathophysiology may prove to be crucial for identifying early interventions that may prevent long-term complications.

One proposed mechanism underlying the development and progression of hypertension involves the role of the vascular cellular environment, particularly the influence of reactive oxygen species (ROS) on vascular function. These byproducts of cellular respiration may influence vascular compliance, tissue remodeling, inflammation, and apoptosis [16,17]. Under normal cellular conditions, cellular compartmentalization and antioxidant defenses exist to mediate the effects of ROS and maintain homeostasis [16,18]. Several studies have indicated that pediatric hypertension results in increased markers of oxidative stress or reduced antioxidant capacity [19,20,21,22,23,24], which may represent the failure of the antioxidant system or an accumulation of ROS, resulting in aberrant cell signaling and function [16,25]. Despite these findings, some studies have failed to establish a direct link between pediatric hypertension and oxidative stress, suggesting instead that ROS accumulation may result from conditions associated with primary hypertension, such as obesity or metabolic syndrome [26,27].

This review will examine the relationship between chronic pediatric hypertension and redox imbalance, with a focus on mechanisms that are relevant to both primary and secondary hypertension. Specifically, we will explore the interplay between the modifiable risk factors contributing to oxidative stress and primary pediatric hypertension, as well as the role of conditions underlying secondary hypertension. By investigating how redox imbalances across various organ systems influence hypertension, we aim to highlight shared mechanistic pathways that could inform future treatment strategies.

## 2. Reactive Oxygen Species

On a cellular level, electrons are harnessed via redox reactions to construct an intracellular reducing environment that is beneficial for cell function. Among other functions, nicotinamide adenine dinucleotide phosphate (NADPH) is utilized by the enzymes glutathione reductase and thioredoxin reductase to maintain cellular levels of reduced glutathione (GSH) and reduced thioredoxin (TRX-SH), which maintain structural integrity within the cells by actively facilitating protein folding, aiding in DNA synthesis and repair, and regulating apoptosis [28,29,30,31,32,33]. Oxidized glutathione and thioredoxin are then recycled back into their reduced states. In this way, NADPH acts as the key reducing currency by which numerous enzymes perform various anabolic processes and provide antioxidant defense.

Another major role performed by the intracellular reducing environment is to neutralize ROS, which include a variety of molecules such as superoxide (O_2_^−^·), hydrogen peroxide (H_2_O_2_), hydroxyl (·OH), and nitrogen-containing species such as nitric oxide (NO·) [34]. ROS represent the products of cellular metabolism and serve central functions in cell signaling. For example, metabolically active cellular compartments, including the peroxisome, plasma membrane, cytosol, and nucleus, participate in nuclear factor erythroid 2-related factor 2 (Nrf2) activation. Nrf2, among other key transcription factors, regulates ROS at the level of gene expression [35,36,37]. If regulation fails and ROS accumulate to uncontrolled amounts, oxidative stress occurs in the form of cellular damage and pathology [38]. In contrast, reductive stress can occur if the reducing environment overcorrects to inhibit the functional utility of ROS [39]. Normal physiology dictates a redox balance between these two states of stress, commonly referred to as redox homeostasis. Table 1 provides an overview of ROS sources and their clinical relevance to pediatric hypertension, which are further explored in the following sections.

## 3. Primary Pediatric Hypertension

Oxidative stress is associated with an array of diseases, depending on the organ system involved. Characteristically, energy-demanding processes like the cardiovascular system are more prone to damage, precipitating hypertension as a notable consequence. Many studies have sought to identify the origin of oxidative stress in pediatric hypertension patients. While diet, physical activity, sleep, and environmental conditions have been identified as factors, obesity may be the strongest predictor of pediatric primary hypertension [40,41,42,43,44]. Despite quantifiable differences in the serum markers of inflammation, NO· synthesis, and oxidative stress between subjects of normal weight and those with obesity, measurable damage to systemic vascular function is not typically present in prepubescent subjects [45,46,47]. Other studies suggest that obese pediatric patients often exhibit a hypertensive “high cardiac output” phenotype, as opposed to the hypertensive “vascular” phenotype seen in adults [48]. Notably, there appear to be protective mechanisms in young people that mitigate oxidative damage to the vasculature [27]. These protective mechanisms maintain the NO· levels of the local environment, which are critical to vasoregulatory mechanisms; however, local tissues may become overwhelmed by oxidative stress, resulting in endothelial dysfunction. A study by Monostori et al. in 2010 and a similar study by Paripović et al. in 2018 suggest that measurable endothelial dysfunction in the blood vessels develops after the onset of high blood pressure [25,49]. Therefore, it is possible that an initial acute insult may be necessary in pediatric populations to drive endovascular oxidative stress and initiate the cascade that ultimately leads to sustained hypertension [19,20,21,46]. Table 2 summarizes these acute insults, or risk factors, which are linked to oxidative stress.

### 3.1. Obesity

The prevalence of pediatric overweight and obesity doubled from 1990 to 2021, with one-third of all children and adolescents globally being predicted to suffer from overweight and obesity by 2050 [50]. Obesity is linked to other cardiovascular risk factors, such as type 2 diabetes mellitus (T2DM) and hyperlipidemia [51,52]. Strong evidence supporting metabolic dysfunction as a major cause of primary hypertension enables a targeted search for the underlying mechanisms of disease [53,54].

While adipose tissue provides structure and regulation for energy storage, it is also recognized as an endocrine organ with a number of signaling molecules. Visceral adipose tissue (VAT) may lead to the increased mitochondrial generation of O_2_^−^· and the upregulation of NADPH oxidases (NOX) [55]. Dysfunctional and excessive VAT is also known to activate a serious inflammatory response, including increased high-sensitivity C-reactive protein, and to inhibit the anti-inflammatory response, including decreased adiponectin levels [56,57]. Regarding treatment, lifestyle intervention remains the preferred method to reduce VAT in children and adolescents [58].

Hyperglycemia is a hallmark of metabolism-associated diseases. A notable consequence of hyperglycemia is the formation of advanced glycation end-products (AGEs), which are important factors for pathogenesis in the pediatric population [59]. AGEs are the combination of a reducing sugar and other proteins, lipids, or nucleic acids that accumulate extracellularly and bind to the receptor for AGE (RAGE). Upon binding, RAGE is known to activate NOX and induce mitochondrial dysfunction and is associated with the oxidative stress markers 8-hydroxy-2′-deoxyguanosine and acrolein-lysine. RAGE also upregulates nuclear factor-kB and TNF-α expression, further activating NOX [60,61]. The resulting oxidative stress may decrease NO· functionality through peroxynitrite formation, leading to vascular dysfunction. Dietary intervention and glycemic control remain a vital preventative measure to limit AGEs and the associated complications [62]. RAGE antagonists have also sparked an interest in limiting endothelial damage [63].

In healthy children, insulin plays a key part in glycemic control while also stimulating vasodilation. Insulin acts on its receptor, which is located on endothelial cells to trigger the activation of phosphatidylinositol 3-kinase (PI3K) and Akt, which are responsible for the phosphorylation of endothelial eNOS at Ser1177, leading to subsequent NO· production [64,65]. In addition to PI3K/Akt/eNOS, insulin also activates Ras and the mitogen-activated protein kinase (MAPK) cascade, increasing endothelin-1 (ET-1) synthesis, which is important in vasoconstriction [66]. Insulin-resistant children show increased levels of the oxidative stress markers nitrite and nitrate, and decreased levels of the antioxidant glutathione peroxidase [67]. Taken together, the effects of oxidative stress on ET-1 activation in insulin-resistant children remain a topic of interest for future studies. Later stages of metabolic dysregulation in young people show the characteristics of pancreatic beta cell dysfunction, further reducing the protective effects of insulin on the vasculature [68,69]. To illustrate, in their study, Gehrmann, Elsner, and Lenzen discussed ROS generation in lipotoxicity within beta cells [70]. The mitochondrial oxidation of non-esterified fatty acids (NEFAs) generates reducing equivalents for ATP production and is associated with H_2_O_2_ accumulation [71,72,73]. Thioredoxin plays an important role in regulating H_2_O_2_ levels inside beta cells [74]. However, there exists a lack of sufficient antioxidants in pancreatic beta cells, facilitating a specific vulnerability to oxidative stress [75]. In this instance, heightened H_2_O_2_ levels activate protein kinases like endoplasmic reticulum kinase (PERK), followed by the subsequent phosphorylation of activating transcription factor 4 (ATF4), resulting in the upregulation of C/EBP homologous protein (CHOP), which is known to initiate cell apoptosis through various mechanisms [76]. Interestingly, vitamin D may decrease apoptosis from oxidative stress through the inhibition of PERK/ATF4/CHOP, showing potential for therapeutic use [77]. The glucagon-like peptide 1 receptor agonist Liraglutide also improves beta cell function in young people, as well as lowering oxidative stress and low-grade inflammation [78].

### 3.2. Other Primary Sources of Oxidative Stress

While oxidative stress is a natural byproduct of cellular metabolism, its burden can be significantly influenced by external factors. In children, their lifestyle habits and environmental exposures play a critical role in shaping oxidative stress levels, which, in turn, impact long-term cardiovascular and metabolic health. Diet, physical activity, environmental pollutants, sleep patterns, and even adverse experiences have all been implicated as modulators of oxidative stress.

Over the past 6 centuries, the nutritional value of food has suffered due to various pressures of modernization [79]. Increased sodium intake, a hallmark of modern dietary consumption, is linked to hypertension in children and adolescents [80]. High sodium diets may induce endothelial dysfunction through uncoupling NOX, thereby producing O_2_^−^· instead of NO· [81]. Conversely, episodes of severe acute malnutrition in childhood are associated with hypertension in adulthood [82]. Malnutrition increases oxidative stress in children through a decreased consumption of antioxidants, possibly causing mitochondrial dysfunction [83]. A healthy, antioxidant-rich diet remains a protective measure against oxidative stress in the pediatric population, with benefits seen in as little as two weeks [84].

In addition to diet, physical activity may act as a mechanism for reducing both oxidative stress and blood pressure in children. While physical activity may initially increase ROS, children with regular exercise routines show lower levels of oxidative stress [85,86]. This can be explained by an initial increase in oxygen utilization, followed by the upregulation of antioxidant mechanisms, including the Nrf2 pathway. Additionally, exercise-mediated upregulation in eNOS activity and the downregulation of vascular NOXes may improve endothelial function and serve a protective role against the development of hypertension [87].

Environmental stressors also influence blood pressure in children. Traffic-related air pollutants are associated with increased markers of oxidative stress and blood pressure [43]. Other pollutants that have been proven to have harmful effects on pediatric blood pressure include sulfur dioxide, nitrogen dioxide, ozone, and carbon monoxide [88]. While the oxidative stress induced by these pollutants has mostly been studied in relation to their effects on asthma, other authors discuss the likelihood of underlying air pollution having a role in pediatric hypertension [89,90]. Although these pollutants are thought to reduce the bioavailability of NO· in endothelial cells, further studies are needed to elucidate the mechanisms behind this process.

Sleep affects many aspects of health and, thus, is a critical aspect to consider in the development of pediatric hypertension. Sleep restriction may be associated with decreased Akt signaling, which is important for insulin’s vasodilatory function via PI3K/Akt/eNOS [91]. Additionally, sleep disorders contribute to autonomic nervous system dysfunction and inflammation [92]. For example, obstructive sleep apnea in children is responsible for a decrease in endothelial NO· bioavailability via increased ROS and pro-oxidative inflammatory markers, including IL-1, IL-6, and TNF-α [93].

More recently, research has begun to focus on psychologically traumatic exposure as a source of oxidative stress. Increasing evidence indicates that children who experience various stressors in childhood have higher rates of cardiovascular risk factors, including hypertension [94]. Studies employing adverse childhood experience (ACE) screening tools alongside the urinary or plasma biomarkers of lipid peroxidation have identified trends indicating greater ROS accumulation in ACE-positive (ACE+) individuals, compared to their ACE-negative (ACE−) counterparts [95]. One study examining oxidative stress in young women who were ACE+ found evidence of reduced endothelial function that was not improved by cardiovascular conditioning. These changes were also associated with elevated levels of oxidized low-density lipoproteins, a marker of oxidative damage, which showed a significant relationship with SIRT1, a key regulator of oxidative stress that is known to activate eNOS [96].

## 4. Secondary Pediatric Hypertension

As supported by multiple studies across various populations, the most recent AAP guidelines for the screening and management of pediatric hypertension suggest that secondary hypertension occurs less frequently in the general population and is typically diagnosed in younger individuals, typically those under 6 years of age [1,97,98,99]. Studies indicating higher rates of secondary hypertension are often conducted in the inpatient or tertiary care setting, which is perhaps associated with a higher need for acute and specialized care [100,101,102,103,104]. In younger children, further evaluation is the gold standard to determine whether an underlying pathology contributes to vascular dysfunction, especially in the case of severe acute hypertension [1,11]. In children older than six years of age, additional evaluation is often not pursuedin the presence of obesity or a family history of obesity [1,99].

**Figure 1:**
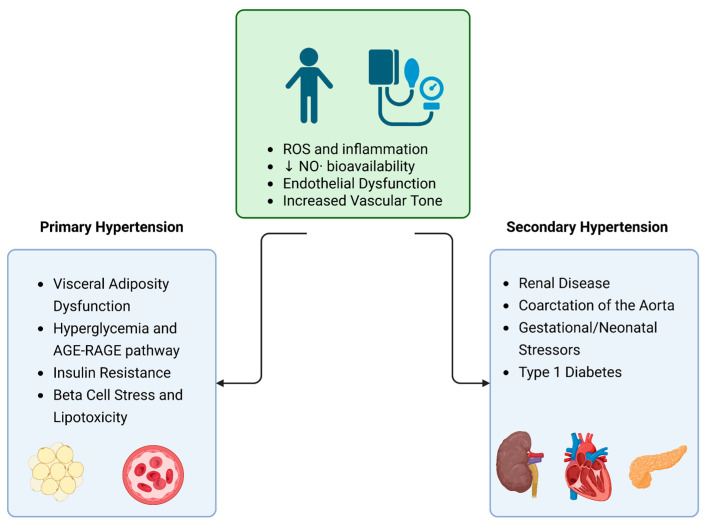
Causes of oxidative stress in primary and secondary pediatric hypertension. Created in BioRender. Backston, K. (2025), https://BioRender.com.

Common causes of secondary hypertension are associated with renal dysfunction, aortic coarctation, gestational or neonatal complications, endocrine disorders, and drug-induced injury [105]. However, there is a paucity of literature detailing how endocrine and pharmacologic mechanisms are independent in terms of their effects on the vasculature. Given the frequent overlap with direct vascular stimulation or renal dysfunction, these etiologies will not be covered in this review. These conditions are typically managed with hormone replacement therapy, surgical tumor resection, antihypertensives, substance withdrawal, or strategies aimed at protecting renal function. One notable exception is type 1 diabetes mellitus (T1DM), which warrants brief consideration due to its distinct oxidative stress-mediated vascular effects. A summary of the causes of both primary and secondary hypertension is depicted in Figure 1.

### 4.1. Renal Disease

Children with renal disease represent the predominant population affected by pediatric secondary hypertension, with hypertension present in 77–97% of cases [99]. Renal disease includes renal parenchymal disease, which comprises glomerulonephropathies, polycystic kidney disease, and, to a lesser extent, congenital structural abnormalities [1,11]. The smaller subset of this population comprises those with renovascular disease [106]. Initiation of the deleterious effects of these various renal pathologies is likely initiated by hypoxia, as demonstrated by Friederich-Persson et al. [107]. The result is chronic kidney disease (CKD), which then progresses to kidney failure.

Throughout its course, CKD presents with varying degrees of uremia, inflammation, and lipid peroxidation. These factors lead to signaling cascades that upregulate both NO· and ROS production. These factors damage the tubular endothelium and activate the renin-angiotensin-aldosterone system (RAAS), with systemic effects. A vicious cycle is created by renal disease with cardiovascular remodeling and subsequent hypertension. Although distinct sources of oxidative stress in renal disease are well-documented, it is important to recognize that the mechanisms outlined below also contribute to primary hypertension, reflecting the widespread influence of cellular metabolism across organ systems.

#### 4.1.1. Uric Acid

In pediatric CKD, the buildup of toxic substances such as uric acid disrupts homeostasis. During purine metabolism, xanthine oxidase generates uric acid, while intermediary conversions produce superoxide radicals and hydrogen peroxide [108]. Extracellularly, uric acid acts as an antioxidant; however, it may also enter the intracellular environment, where it acts as an oxidant in the presence of NOX, increasing ROS, and causing mitochondrial injury and lipid accumulation [109].

Serum uric acid levels have been implicated independently as a predictor of CKD progression [110,111], and, along with its intermediaries, is also associated with primary hypertension in children [112,113]. Additionally, in a study on obese adolescents, serum uric acid was found to be independently associated with the inflammatory markers CRP, IL-6, and sICAM-1 [114]. As varying degrees of uremia are present in most stages of CKD, it is reasonable to conclude that the uremia associated with CKD may impact blood pressure levels due to the production of ROS, the uncoupling of eNOS, and the induction of inflammation.

#### 4.1.2. Lipid Oxidation

In the intracellular environment, lipids subjected to increased oxidative stress will give rise to lipid peroxidases. This is typically mediated by antioxidants like glutathione peroxidases as a first-line defense, supported by the dietary antioxidants α-tocopherol (vitamin E) or carotenoids [115]. Lipid peroxidases and ROS are reactive with the plasma membrane of cells and will interact with circulating lipoproteins resulting in the accumulation of ROS, which freely interact systemically [116,117].

Studies of children with CKD have revealed that even in the early stages of the disease, the high-density lipoproteins (HDLs) isolated from serum samples showed aberrant behavior associated with staging [118,119]. Traditionally a cardioprotective lipoprotein, these abnormal HDLs resulted in reduced NO· production and increased inflammatory signaling with urate, IL-6, and angiopoietin-2 when introduced to healthy endothelial cells in vivo [118]. Another study found similar results when looking at the endothelial response with cells releasing inflammatory markers and reduced proliferative capacity [119]. Moreover, when HDLs were added to macrophages, their chemotactic properties were enhanced through IL-1β, TNF-α, and MCP-1 [119].

When investigating a source for this behavior, researchers found high levels of SDMA, a product of arginine metabolism that results from diminished NO· metabolism and is an indicator of endothelial dysfunction [118]. A prior study by Speer et al. in 2013 found that SDMA in HDLs directly stimulated Toll-like receptor-2 in the endothelium, thereby linking it to immune function, endothelial dysfunction, and the immune system [120].

Oxidation also occurs in low-density lipoproteins (LDLs), resulting in endothelial dysfunction and changes in macrophage behavior. In pediatric patients, oxidized LDLs, while not directly associated with inflammatory markers, were associated with higher blood pressure and left ventricular hypertrophy, which was also associated with CKD staging [121]. Macrophages exposed to oxidized LDLs downregulate the markers of classical inflammation and begin to express alternate activation, which promotes tissue remodeling and fibrosis, with an increased risk of foam cell formation [122,123,124]. Thus, through the competing interests of modified lipoproteins, we can see the complex microenvironment generated by oxidative stress.

#### 4.1.3. Inflammation

Inflammatory responses may be triggered by factors such as changes in shear stress on the vascular wall, cellular death, and the presence of reactive oxygen species (ROS). These triggers promote the release of the cytokines that drive endothelial remodeling, which can lead to dysfunction. While multiple mechanisms contribute to the development of hypertension, inflammation consistently emerges as a central feature. In pediatric populations, findings in the literature often intersect with a range of pathologies linked to both primary and secondary hypertension. This discussion emphasizes the unifying role of inflammation and its impact on the altered physiology of various systems.

A well-characterized inflammatory cytokine implicated in both renal disease, hypertension, and oxidative stress is IL-6. In vitro and murine studies suggest that IL-6 induces endothelial dysfunction via the angiotensin II type 1 receptor, which, similarly to uric acid, stimulates NADPH oxidase and increases ROS production [125]. In addition, IL-6 is also known to promote the recruitment and maturation of immune cells and increase adhesion molecule expression on the endothelium, thus supporting the immune cell infiltration of tissue [126].

Several pediatric studies have shown a relationship between the progression of chronic kidney disease (CKD) from multiple origins and the activation of monocyte/macrophage stimulation and migratory chemokines [127,128,129,130,131]. Macrophages exhibit varying phenotypes, depending on the stage of disease and the local environment within which they are activated. In classic inflammation, they tend to adopt a pro-apoptotic phenotype, in which damaged cells and the associated oxidized LDLs are phagocytosed [132]. In vitro studies using human macrophages have demonstrated that ROS, generated by NADPH oxidase, enhance the differentiation of monocytes to pro-apoptotic macrophages and decrease COX-2 expression, thereby perpetuating the inflammatory cycle [133]. This dysfunction leads to the formation of foam cells, which aggregate to form arterial plaques [134]. Mouse models have shown mild decreases in renal function to be associated with foam cell formation, which was stimulated by the inflammatory stimulus NF-κB [135]. The growth of arterial plaque results in localized necrosis and continuous remodeling of the vasculature, as well as turbulent flow [134].

T cells are also implicated in vascular inflammation. A recent study found the presence of cytotoxic granzyme B and perforin-1 at significant levels in the serum of hypertensive children. The genes associated with the transcription of these proteins were significantly correlated with systolic blood pressure (SBP) [136]. These findings are congruent with the observation that an impaired T cell balance exists in hypertensive patients. Regulatory T cells (Tregs) may be the most affected and are suspected to be a result of elevated levels of IL-6 promoting Th17 maturation [132]. In both mouse models and adults, Th17 is implicated in hypertension, as well as superoxide production [137,138]. Moreover, a study on obesity and the Th17/Treg balance confirmed that this relationship was more strongly associated with blood pressure, as opposed to metabolic status [139].

Both ROS and inflammation are central to the development and progression of hypertension, with key roles being played by cytokines like IL-6, macrophages, and T cells. These factors interact in a vicious cycle, promoting endothelial dysfunction, foam cell formation, and vascular remodeling. Further research is needed to better understand the mechanisms by which inflammation and ROS contribute to pediatric hypertension, particularly regarding the roles of Th17 cells and the balance between immune cell subsets.

#### 4.1.4. Nitric Oxide Balance and Asymmetric Dimethylarginine

Asymmetric dimethylarginine (ADMA) is generated via proteolysis in endothelial cells, forming citrulline, which is used to regenerate arginine, and the waste product dimethylamine (DMA) [140,141]. ADMA has been extensively studied over the past three decades and is well-characterized in the literature. Elevated ADMA levels can significantly increase systemic vascular resistance by inhibiting the production of arginine-derived endothelial nitric oxide synthase (eNOS) [142]. Moreover, elevated levels of ADMA and its associated metabolites have been linked to renal insufficiency and are closely associated with the staging of renal dysfunction [140]. ADMA is thought to reflect the systemic endothelial dysfunction caused by oxidative stress and is also reflective of the cardiovascular risk associated with CKD progression [140,143]. While these mechanisms are well-established in adult populations and animal models, their relevance in pediatric hypertension remains underexplored.

Recent pediatric studies suggest that disruptions to ADMA metabolism and the resulting impairment in NO· synthesis may contribute to vascular dysfunction in children with renal disease. Low urinary and high plasma ADMA levels have been observed across various renal conditions in pediatric populations [144,145,146,147]. In polycystic kidney disease, elevated serum concentrations of methylated products of the urea and NO· cycle, specifically ADMA and SDMA levels, were associated with disease severity [148,149]. Thrombomodulin, a glycoprotein that is traditionally associated with cardiovascular disease, has also been found to be correlated with elevated serum ADMA levels in children with poor renal function [150]. Children with chronic kidney disease and blood pressure abnormalities have lower urinary citrulline-to-arginine ratios but higher plasma levels, with urine markers that are linked to vascular remodeling and plasma levels correlating with blood pressure [145,146]. As many children in these studies had hidden hypertension that was detectable via 24-h ambulatory blood pressure monitoring, it was suggested that the elevation of citrulline in the plasma was a compensatory mechanism of the pre-hypertensive state [146]. Additionally, plasma SDMA, an inactive isomer of ADMA that limits NO·NO production through its consumption of arginine, is solely excreted by the kidney and may be a strong marker for diminished kidney function and blood pressure variability [142]. More recent studies have also suggested that SDMA, initially thought to be biologically inactive, is involved in the resulting local and systemic endothelial dysfunction, as previously discussed [119,150]. Finally, ADMA has also been linked to hypertensive patients with IR and obesity, suggesting its relevance in both primary and secondary forms of hypertension [151,152]. Larger clinical studies or meta-analyses may help clarify its role across diverse etiologies and bridge the existing gaps in our understanding of hypertension in pediatric populations.

### 4.2. Coarctation of the Aorta

Coarctation of the aorta accounts for approximately 4–8% of all congenital heart defects and is commonly associated with a bicuspid aortic valve [153,154]. While frequently asymptomatic in the neonatal and adolescent periods, the condition is typically diagnosed in childhood in the context of elevated blood pressure [154]. Its impact on vascular responsiveness to oxidative stress made it a compelling model in early translational models. Specifically, early rodent models demonstrated increased levels of NADPH oxidase, superoxide dismutase (SOD), and NO·-ROS intermediates, which is suggestive of increased oxidation [155,156]. These findings suggest that shear stress on the vascular wall is a potent stimulator of oxidative stress and contributes to the development of hypertension.

Treatment modalities for coarctation repair have also been implicated in oxidative stress, an area that will be explored in subsequent sections. Despite its clinical relevance to pediatric hypertension and the results of preclinical models, few studies have focused exclusively on the effects of aortic coarctation in pediatric populations. Notably, a study by Skeffington et al. compared the proteomic profiles of pediatric patients with isolated coarctation against those with a coexisting bicuspid aortic valve [157]. Patients with both anomalies demonstrated statistically significant elevations in the proinflammatory markers involved in macrophage recruitment and activity, which we have previously discussed as impacting arterial pressure and SOD [157].

### 4.3. Gestational and Neonatal Stressors

Fetal and neonatal stressors are common and affect roughly 10.4% of US pregnancies and 10% of pregnancies worldwide [158,159]. A strong correlation exists between premature births or preterm births and pre-eclampsia, which causes fetal growth restriction, the most susceptible fetal organ that is affected being the kidneys [160,161]. The primary concerns surrounding premature labor and pre-eclampsia are maternal and infant mortality. Beyond these immediate risks, both conditions have been linked to increased oxidative stress in the neonate, as well as a maternal contribution to oxidative stress burden during fetal development. In addition to this, epidemiologic studies have demonstrated a link between preterm birth and developing hypertension [162,163].

Preterm infants requiring admission to neonatal intensive care units exhibit decreased levels of circulating antioxidants and elevated markers of oxidative stress, these being associated with acute complications like bronchopulmonary dysplasia and intraventricular hemorrhage [164]. Additionally, cord blood collected following the delivery of infants born to hypertensive, pre-eclamptic mothers demonstrated elevated malondialdehyde (MDA), a byproduct of lipid peroxidation and a recognized biomarker of oxidative stress [161].

These systemic effects also appear to have long-lasting consequences. A study by Jayet et al. demonstrated abnormalities in pulmonary and systemic arterial vascular pressures among adolescent children born to preeclamptic mothers, exhibiting significant differences when compared to both control patients and related siblings of normotensive pregnancy [165]. These vascular changes were found to be associated with increased plasma concentrations of lipid peroxidation, as measured by thiobarbituric acid-reactive substances (TBARS) [165]. Moreover, there are multiple cardiovascular risk factors that adults who were born prematurely will experience at higher rates than adults with normal gestation periods, namely, hypertension, obesity, and glucose intolerance [166]. However, associating these factors with one another has not proved to be significant between study and control groups, encouraging further investigation into cellular inflammation and oxidative stress as key players in contributing to these pathologies [166].

A compelling extension of this concept is the association between assisted reproductive technology (ART) and the development of hypertension in offspring. Multiple studies have demonstrated that, compared to children conceived through natural fertilization, ART-conceived children have higher rates of hypertension, a finding that may be linked to changes in nitric oxide synthase, oxidative stress, and endothelial dysfunction [167,168,169]. While the mechanism behind this finding is unclear, it suggests that the environment is critical to the development and function of the vasculature, even at the earliest stages. Epigenetic modifications, oxidative stress during early embryogenesis, and impaired placental function have all been proposed as potential contributors. These findings highlight the need to further investigate prenatal influences on long-term cardiovascular health and reinforce the concept that the origins of hypertension may begin as early as conception.

### 4.4. Type 1 Diabetes Mellitus

The systolic blood pressure changes observed in insulin-resistant T2DM are thought to be related to modifiable risk factors such as obesity. In contrast, type 1 diabetes mellitus (T1DM) represents an independent secondary cause of pediatric hypertension. This is particularly notable in the context of renal disease, as T1DM can affect both the renal parenchyma and the renal vasculature. We have discussed these mechanisms at length as they relate to oxidative stress and pediatric hypertension. However, there are other mechanisms implicated in the development of hypertension in T1DM, which affects 6–16% of the pediatric population [170].

Among those patients with T1DM is a subpopulation without diabetic kidney disease despite years of disease who develop essential hypertension, suggesting an alternative mechanism for the resulting arterial dysfunction [171]. Increased arterial stiffness in T1DM subjects without an existing diagnosis of hypertension or CAD may be caused by a significant elevation in oxidized LDLs [21,172]. This supports the implication that oxidative stress is associated with vascular changes in this population. Additionally, there appears to be a diminished antioxidant defense system in pediatric T1DM patients, and compounding risk factors such as metabolic syndrome contribute to disease progression and blood pressure changes [172,173].

As discussed within the setting of insulin resistance and T2DM, T1DM is also associated with the accumulation of AGEs. In adults, there is an established relationship between AGEs, oxidative stress, inflammation, and endothelial dysfunction. The initial studies involving children implicated AGEs as a source of the microvascular changes contributing to diabetic kidney disease or hypertension [174]. While AGEs accumulate at higher rates in diabetic children [175], more recent studies have been unable to establish them as an independent predictor of diabetic kidney disease or systemic vascular dysfunction [176,177]. However, measurement methods vary across such studies, which may reflect either differences in sampling sources or, perhaps, the reduced extent of cellular senescence in children.

## 5. The Influence of ROS on the Treatment of Hypertension

**Figure 2:**
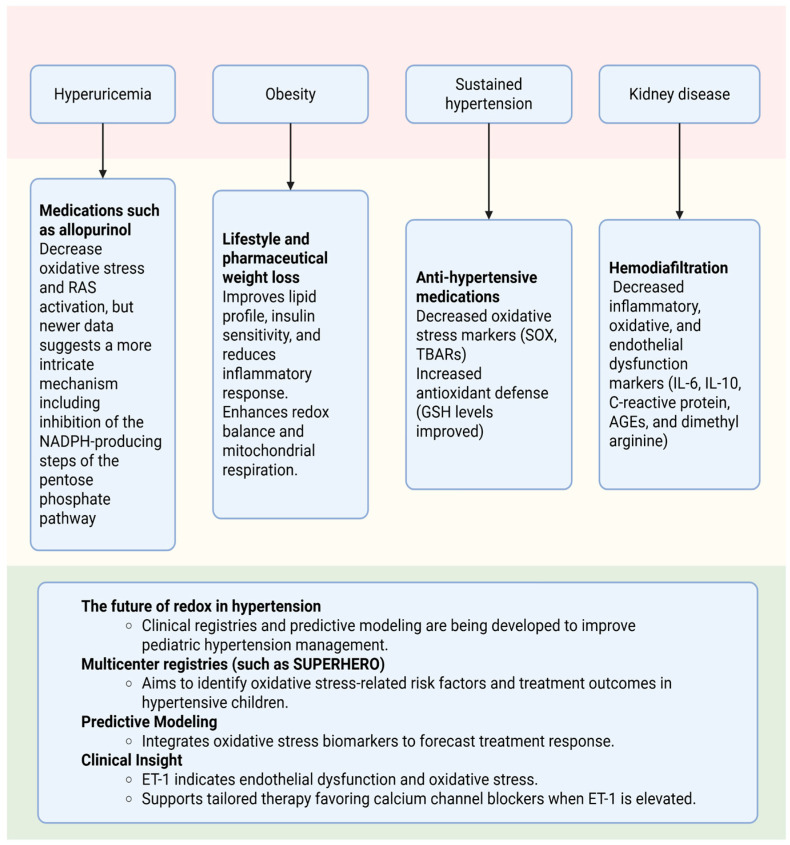
Common interventions for redox imbalance in pediatric hypertension and other comorbidities.

Given the central role of oxidative stress in the pathogenesis of pediatric hypertension, its influence must be carefully considered when evaluating the efficacy and long-term implications of both medical and procedural interventions. As key mediators of endothelial dysfunction and vascular remodeling, ROS have the potential to modulate not only the disease trajectory but also the therapeutic response. Understanding these redox-sensitive pathways is critical to optimizing existing treatments and guiding the development of targeted, data-driven strategies in pediatric hypertension management. Figure 2 provides a synopsis of the interventions for redox imbalance and other tools that may support treatment in the future.

Medical treatments for the discussed conditions should be evaluated not only for their intended therapeutic effects but also for their impact on oxidative stress and blood pressure. As notable examples in the context of obesity, phentermine and topiramate gained FDA approval in 2022 for treating pediatric patients aged 12 and older who are obese [178]. Recent data from a phase 4 trial concluded that phentermine and topiramate had beneficial effects on blood pressure, resulting in the removal of the hypertension warning from its label [179]. Despite this news, phentermine’s sympathomimetic properties, potentially causing ROS generation, should be carefully considered and require further investigation.

In the context of sustained hypertension, several pharmacologic studies have examined the impact of treatment on oxidative stress. In a study in which children were prescribed an ACE (or ARB, in the case of ACE intolerance), patients showed decreases in the oxidative stress biomarkers SOX and TBARs and also an improved antioxidant defense, as demonstrated by increased GSH levels. However, these findings were not directly correlated to a decrease in blood pressure [26]. Further investigation is needed to determine how the modulation of oxidative stress influences long-term clinical outcomes in hypertensive children, along with the best measure of vascular pressure to evaluate pharmacologic efficacy.

Other hypertensive treatments have been linked to a decrease in oxidative stress. Reducing uric acid levels with allopurinol has gained traction as a pediatric hypertensive therapy in recent years [180,181]. Treatment with allopurinol is thought to decrease oxidative stress and RAS activation, but newer data suggest a more intricate mechanism, including inhibition of the NADPH-producing steps of the pentose phosphate pathway [182]. Additional research is required in order to clarify the overall effects of allopurinol on oxidative stress and its role in treating pediatric hypertension.

Procedural and surgical interventions to address the conditions contributing to hypertension also present oxidative stress challenges. In children with kidney failure requiring dialysis or kidney transplantation, oxidative stress and hypertension also exist as side effects of their treatment [183]. These patients display lower glutathione and higher C-reactive protein levels, with correlated cardiac dysfunction [184]. In order to minimize oxidative damage, certain therapies may be considered over others. Hemodiafiltration is reported to produce fewer inflammatory, oxidative, and endothelial dysfunction markers (IL-6, IL-10, C-reactive protein, AGEs, and dimethyl arginine, among others) compared to high-flux hemodialysis [185].

Unexpected oxidative effects have also been observed following cardiac procedures. In a sheep model of aortic coarctation, stent placement led to increased oxidative stress and inflammation, measured by SOD1, NOS3, and MMP3 at the stent site one year post-operatively, despite the resolution of hypertension and a technically successful procedure [186]. Similarly, in an observational study by Martins et al., children and young adults who underwent coarctation repair with stenting, balloon dilation, or surgical repair were evaluated at least 6 months after treatment. While all groups showed similar mortality, balloon dilation was associated with greater vascular compliance but also with the highest levels of inflammatory and macrophage-mediated tissue remodeling markers [187]. Collectively, these findings suggest that improved hemodynamics may coexist with persistent oxidative stress and tissue remodeling, highlighting the complexity of treatment outcomes and the need for further research to understand the implications of these findings.

Emerging tools such as clinical registries and predictive modeling are being developed to better capture long-term outcomes and personalize treatment. Data on the cardiovascular risk burden of pediatric hypertension and long-term outcomes remain sparse and ill-defined. To address this, Atrium Health Levine Children’s Hospital in North Carolina and 13 other sites across North America have begun creating the SUPERHERO registry. This de-identified database aims to collect relevant laboratory and demographic data from children diagnosed with hypertension to better define the associated risks and treatment outcomes [188]. From this ambitious project, future research may be able to clarify the role of oxidative stress in patient outcomes and thereby guide treatment.

On a clinical front, predictive modeling is beginning to incorporate oxidative stress biomarkers as variables to forecast medication response. In a model evaluating amlodipine efficacy, elevated levels of endothelin-1 (ET-1) were associated with greater responsiveness to treatment. ET-1 elevations are associated with endothelium dysfunction and increases in oxidative stress, due to the activation of intracellular calcium influx. In contrast, patients with insulin resistance or hyperinsulinemia demonstrated the reduced efficacy of amlodipine, suggesting alternative oxidative pathways that are not targeted by this drug [189]. Regarding other recent biomarker studies, the systemic immune-inflammation index, neutrophil-to-lymphocyte ratio, and platelet-to-lymphocyte ratio are positively correlated with hypertension, while the lymphocyte-to-monocyte ratio is negatively correlated with hypertension in pediatric Americans [190]. IL-2 is also lower in hypertensive children [191].

## 6. Conclusions

Pediatric hypertension represents a multifaceted disorder shaped by the intersection of metabolic, developmental, and environmental factors, with oxidative stress emerging as a central mechanistic link across diverse etiologies. The disruption of redox homeostasis not only initiates endothelial dysfunction but also amplifies vascular injury through chronic inflammation, immune dysregulation, and impaired nitric oxide signaling. Increasing evidence supports the notion that redox imbalance is not merely a byproduct of disease but is instead a fundamental driver of its pathogenesis and progression. As the burden of hypertension rises in younger populations, a deeper molecular understanding of redox signaling across organ systems will be essential to inform risk stratification, guide therapeutic innovation, and improve long-term cardiovascular outcomes. While large cohort studies remain a necessity for clinical guidance, future research must harness the growing technological advancements in machine learning. In parallel, the continued development of pediatric biorepositories is essential for machine learning algorithms to discover novel redox biomarkers that remain hidden with traditional methods. Integrating this data-driven approach and redox biology into pediatric hypertension frameworks may redefine both diagnostic and therapeutic paradigms for the next generation of patients.

## Figures and Tables

**Table 1:** Sources of reactive oxygen species related to pediatric hypertension and common comorbidities.

ROS Source	Principal Enzyme (s) or Complexes	Pathophysiologic Contexts in Pediatrics	Molecular Mechanism of ROS Generation	Downstream Vascular Effects
Mitochondrial Electron Leakage	Electron transport chain Cytochrome c redox cycling	Obesity Insulin resistance CKD Type 1 Diabetes mellitus	Hyperglycemia and excess substrate influx overstimulate mitochondrial metabolism Generation leads to electron leakage and the partial reduction of O_2_ to superoxide (O_2_^−^·)	Mitochondrial oxidative damage Apoptosis of endothelial cells Impaired ATP synthesis
NADPH Oxidase Activation	Adipokines and pro-inflammatory cytokines Leptin Interleukin-1b Interleukin-6 TNF-α Monocyte chemoattractant protein-1 DAG activation of protein kinase C AGEs Uric acid	Obesity Insulin resistance Visceral adipose tissue inflammation Renal disease	Cytokine-mediated NOX assembly on plasma membrane catalyzes the one-electron reduction of O_2_ to O_2_^−^·	Uncoupling of endothelial nitric oxide synthase Increased vascular tone Immune cell recruitment
Xanthine Oxidoreductase System	Uric acid	Uremia in CKD Hyperuricemia Low-grade chronic inflammation	Transition of xanthine dehydrogenase to oxidase form (XDH → XO) facilitates ROS production (O_2_^−^·, H_2_O_2_) during purine catabolism and ischemia-reperfusion	Endothelial injury Lipid peroxidation Activation of renin-angiotensin system
AGE-RAGE Signaling Axis	Advanced glycation end products Receptor for AGE (RAGE) NF-κB Protein Kinase C	Obesity Insulin resistance CKD Type 1 Diabetes mellitus	Non-enzymatic glycation of proteins/lipids forms AGEs AGEs bind RAGE to activate NF-κB ↑ NOX transcription, ↑ cytokines (IL-6, TNF-α), and protein kinase C signaling	Pro-inflammatory endothelial phenotype Increased vascular permeability ROS-NO· imbalance
Peroxisomal β-Oxidation	Acyl-Coenzyme-A Oxidase Catalase	Fatty acid overload Insulin resistance Perinatal lipotoxic stress	Very-long-chain fatty acids undergo peroxisomal β-oxidation producing H_2_O_2_ Excess overwhelms catalase defense Exacerbated by insufficient antioxidant systems in children	Oxidative β-cell stress Endothelial stress Impaired redox buffering
Arginine Methylation Pathway	Asymmetric Dimethylarginine (ADMA) Symmetric Dimethylarginine (SDMA) Dimethylarginine Dimethylaminohydrolase (DDAH)	CKD Polycystic kidney disease Systemic endothelial dysfunction	ADMA/SDMA competitively inhibits eNOS Angiotensin II suppresses DDAH-2 expression This leads to ADMA accumulation and nitric oxide depletion	Vasoconstriction, endothelial dysfunction Impaired flow-mediated dilation Exacerbated blood pressure variability

**Table 2:** Risk factors for oxidative stress in pediatric hypertension.

Category	Risk Factor	Mechanism of Oxidative Stress	Associated Pediatric Conditions	Clinical Relevance
Metabolic	Obesity	Increased adiposity → visceral fat accumulation → mitochondrial overload and NADPH oxidase activation → ↑ superoxide and hydrogen peroxide production	Primary hypertension, metabolic syndrome, and insulin resistance	Drives ROS generation through excess nutrient metabolism and adipokine-mediated inflammation
	Hyperglycemia	Glucose overload → ↑ mitochondrial respiration + ↑ diacylglycerol → PKC activation → NOX stimulation → ↑ ROS; AGE-RAGE signaling	Type 1 and Type 2 diabetes mellitus	Promotes endothelial dysfunction, protein glycation, and vascular remodeling
	Dyslipidemia	Oxidized LDL and abnormal HDL profiles → foam cell formation → ROS amplification and vascular inflammation	Obesity, CKD, and early cardiovascular disease	Alters lipid metabolism, enhances inflammatory responses, and impairs NO· signaling
Dietary	High sodium intake	Endothelial sodium overload → NOX uncoupling → ROS production, instead, of nitric oxide	Primary hypertension	Increases vascular tone, reduces NO· bioavailability, linked to BP elevation
	Micronutrient deficiency	Insufficient antioxidants (e.g., vitamins and E, and selenium) → impaired ROS neutralization	Malnutrition, stunting, and early-life growth restriction	Reduces redox buffering capacity, increasing vulnerability to oxidative insults
Environmental	Air pollution (PM2.5, NO_2_, O_3_)	Inhaled particulates activate pulmonary macrophages → systemic cytokine release → endothelial ROS; direct ROS induction in lung and vasculature	Urban-dwelling children showing asthma and hypertension	Promotes systemic inflammation and vascular dysfunction
	Tobacco smoke exposure (prenatal/secondhand)	Nicotine and free radicals → oxidative DNA damage, reduced antioxidant enzymes	Preterm birth, SIDS, and hypertension	Impairs fetal and pediatric vascular development
Behavioral/Lifestyle	Physical inactivity	↓ Mitochondrial biogenesis and antioxidant defense (e.g., Nrf2 pathway); ↑ basal inflammation	Obesity and metabolic syndrome	Diminishes adaptive redox response, worsens vascular stiffness
	Sleep deprivation or apnea	Intermittent hypoxia → ↑ ROS via NOX and mitochondrial pathways; ↑ IL-6, TNF-α	Pediatric obstructive sleep apnea	Links to BP elevation, endothelial dysfunction, and sympathetic overactivity
Developmental	Prematurity	Immature antioxidant systems + high oxygen exposure → ↑ lipid peroxidation and ROS	Bronchopulmonary dysplasia and early-onset hypertension	Contributes to long-term vascular programming and oxidative damage
	Pre-eclampsia exposure (in utero)	Placental oxidative stress → fetal endothelial dysfunction + altered NO· signaling	Neonatal hypertension and later-life cardiometabolic risk	Early-life ROS exposure programs hypertension risk
Psychosocial	Adverse childhood experiences (ACEs)	Chronic stress → ↑ cortisol and sympathetic tone → mitochondrial dysfunction + ↑ NOX activity	Hypertension, anxiety, and insulin resistance	Elevates systemic oxidative stress markers and accelerates vascular aging

Causes, decreases, and increases for →, ↓, and ↑.
